# Host A-to-I RNA editing signatures in intracellular bacterial and single-strand RNA viral infections

**DOI:** 10.3389/fimmu.2023.1121096

**Published:** 2023-04-04

**Authors:** Zhi-Yuan Wei, Zhi-Xin Wang, Jia-Huan Li, Yan-Shuo Wen, Di Gao, Shou-Yue Xia, Yu-Ning Li, Xu-Bin Pan, Yan-Shan Liu, Yun-Yun Jin, Jian-Huan Chen

**Affiliations:** ^1^Laboratory of Genomic and Precision Medicine, Wuxi School of Medicine, Jiangnan University, Wuxi, Jiangsu, China; ^2^Joint Primate Research Center for Chronic Diseases, Institute of Zoology of Guangdong Academy of Science, Jiangnan University, Wuxi, Jiangsu, China; ^3^Jiangnan University Brain Institute, Wuxi, Jiangsu, China; ^4^Department of Pediatric Laboratory, Wuxi Children’s Hospital, Wuxi, Jiangsu, China

**Keywords:** A-to-I RNA editing, bacterial infection, viral infection, intracellular bacterial pathogens, pneumonia

## Abstract

**Background:**

Microbial infection is accompanied by remodeling of the host transcriptome. Involvement of A-to-I RNA editing has been reported during viral infection but remains to be elucidated during intracellular bacterial infections.

**Results:**

Herein we analyzed A-to-I RNA editing during intracellular bacterial infections based on 18 RNA-Seq datasets of 210 mouse samples involving 7 tissue types and 8 intracellular bacterial pathogens (IBPs), and identified a consensus signature of RNA editing for IBP infections, mainly involving neutrophil-mediated innate immunity and lipid metabolism. Further comparison of host RNA editing patterns revealed remarkable similarities between pneumonia caused by IBPs and single-strand RNA (ssRNA) viruses, such as altered editing enzyme expression, editing site numbers, and levels. In addition, functional enrichment analysis of genes with RNA editing highlighted that the Rab GTPase family played a common and vital role in the host immune response to IBP and ssRNA viral infections, which was indicated by the consistent up-regulated RNA editing of Ras-related protein Rab27a. Nevertheless, dramatic differences between IBP and viral infections were also observed, and clearly distinguished the two types of intracellular infections.

**Conclusion:**

Our study showed transcriptome-wide host A-to-I RNA editing alteration during IBP and ssRNA viral infections. By identifying and comparing consensus signatures of host A-to-I RNA editing, our analysis implicates the importance of host A-to-I RNA editing during these infections and provides new insights into the diagnosis and treatment of infectious diseases.

## Introduction

The world has recently witnessed the threat that infectious diseases pose to public health, particularly the COVID-19 pandemic caused by SARS-COV-2 (1). These infections are typically caused by pathogenic microorganisms, such as bacteria and viruses (2, 3). In the presence of infection, the innate immunity of infected cells triggers a swift defense response, characterized by the immediate activation of interferons (IFNs), toll-like receptors (TLRs), and the NF-κB pathway (4–8), which in turn contributes to the development of the diseases (9, 10). Recently emerging studies have implicated that similar mechanisms involving the reprogramming of host cell metabolism may be involved in both bacterial and viral infections, owing to the common need for suitable host cells to enable effective replication and proliferation (8).

Epigenetics plays a pivotal role in the context of bacterial and viral infections. Notably, the severity of COVID-19 exhibits a correlation with DNA methylation in genes that are associated with the innate immune response (11). Furthermore, infection by *Streptococcus pneumonia* triggers histone H3 dephosphorylation (12). A-to-I RNA editing, an epigenetic process that converts adenosine (A) to inosine (I) mediated by the adenosine deaminase acting on the RNA (*Adars*) family (13), has been reported to be involved in immune-related diseases and infections (14). Notably, ADAR-mediated A-to-I RNA editing has been reported as a key regulator of innate immune activation and antiviral activities during viral infections (15, 16). RNA editing has also been detected during intra-host evolution in SARS-CoV-2 prolonged infections (17), and infection by polyomavirus and different subtypes of influenza A viruses in epithelial cells (18). Up-regulation A-to-I RNA editing in human epithelial and endothelial cells was reported in *Candida albicans* infection. (19). Although existing studies have reported the important function of RNA editing (20–22), its role in mammalian hosts during bacterial infections has yet to be elucidated.

Herein we conducted a transcriptome-wide analysis of RNA editing profiles of intracellular bacterial pathogen (IBP) infections in various mouse tissues and organs to characterize the consensus signature of host RNA editing. In particular, we compared the host RNA editing patterns between pneumonia caused by IBPs and single-strand RNA (ssRNA) viruses, highlighting both similarities and differences between the two types of infections. Our findings could provide insights into the epigenetic underpinnings of these infectious diseases.

## Results

### Altered A-to-I RNA editing profiles during IBP infections

**Figure 1:**
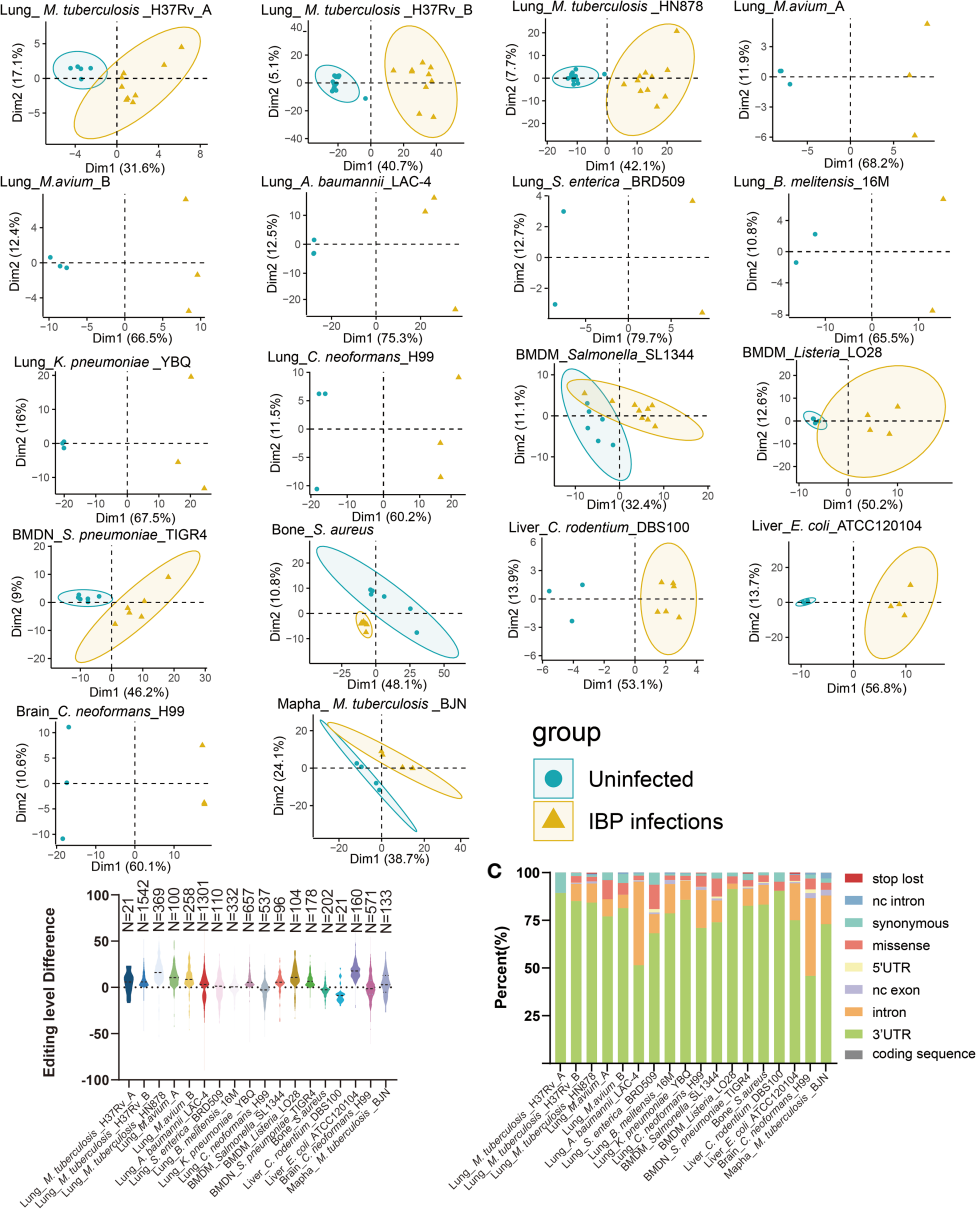
Altered A-to-I RNA editing profiles during IBP infections **(A)** First two principal components of differential editing profiles reveal the difference in RNA editing patterns between the uninfected and infected groups. **(B)** The difference in editing levels between infected and uninfected samples in IBP infections. The number of DRE sites is listed at the top of the plots. **(C)** The functional categories of DRE sites in IBP infections. nc intron: non coding transcript intron variant, nc exon: non coding transcript exon variant.

18 Datasets of IBP infections in mice were analyzed including the lung, liver, right femur, brain, bone-marrow-derived macrophages (BMDM), bone-marrow-derived neutrophils (BMDN), and macrophage cell line raw264.7. All samples from the datasets were firstly combined into an uninfected group and an infected group (**Table 1**) and subjected to subsequent analysis. Principal component analysis (PCA) showed that the uninfected and infected groups clustered separately based on the editing level of differential RNA editing (DRE) sites (**Figure 1A**). In terms of the editing level of the DRE sites, most models (13/18) showed higher editing levels after bacterial infection (**Figure 1B**). The majority of DRE sites in bacterial infections were 3′ -untranslated region (UTR), intronic, and missense variants (**Figure 1C**). These results suggested distinct alteration of RNA editing profiles during IBP infections.

**Table 1:** Details of the GEO datasets included in the current study.

NO.	Bacterial or viral pathogen	Tissue	Abbreviation	Uninfected (N = 66)	Infected (N = 144)	BioProject Accession	Contributors	Citation
*1*	*Mycobacterium tuberculosis* H37Rv	Lung	Lung_ *M. tuberculosis* _H37Rv_A	5	9	PRJNA707548	Naqvi, et al., 2021	(23)
*2*	*Mycobacterium tuberculosis* H37Rv	Lung	Lung_ *M. tuberculosis* _H37Rv_B	15	10	PRJNA564540	Moreira-Teixeira, et al., 2020	(24)
*3*	*Mycobacterium tuberculosis* HN878	Lung	Lung_*M. tuberculosis* _HN878	15	10	PRJNA564540	Moreira-Teixeira, et al., 2020	(24)
*4*	*Mycobacterium avium* subspecies hominissuis	Lung	Lung_*M.avium*_A	3	3	PRJNA603273	Nakajima, et al., 2021	(25)
*5*	*Mycobacterium avium* subspecies hominissuis	Lung	Lung_*M.avium*_B	3	3	PRJNA715641	Nakajima, et al., 2021	(26)
*6*	*Acinetobacter baumannii* LAC-4	Lung	Lung_*A. baumannii*_LAC-4	3	3	PRJNA600998	Zeng, et al., 2020	(27)
*7*	*Salmonella enterica* ser. Typhimurium BRD509	Lung	Lung_*S. enterica* _BRD509	2	2	PRJNA608200	Drashansky, et al., 2021	(28)
*8*	*Brucella melitensis* 16M	Lung	Lung_*B. melitensis*_16M	2	2	PRJNA749252	Demars, et al., 2021	(29)
*9*	*Klebsiella pneumoniae* clinical strain YBQ	Lung	Lung_*K. pneumonia* _YBQ	3	3	PRJNA718245	Zou, et al., 2021	(30)
*10*	*Cryptococcus neoformans* var. grubii H99	Lung	Lung_*C. neoformans*_H99	3	3	PRJNA506308	Li, et al., 2019	(31)
*11*	*Salmonella enterica* subsp. Enterica serovar Typhimurium SL1344	Bone-marrow derived macrophages	BMDM_*Salmonella*_SL1344	6	9	PRJNA413814	Stapels, et al., 2018	(32)
*12*	*Listeria monocytogenes* strain LO28	Bone-marrow derived macrophages	BMDM_*Listeria*_LO28	4	4	PRJNA342315	Szappanos, et al., 2018	(33)
*13*	*Streptococcus pneumoniae* strain TIGR4	Bone-marrow derived neutrophils	BMDN_*S. pneumoniae*_TIGR4	6	6	PRJNA633715	Bhalla et al. 2021	(34)
*14*	*Staphylococcus aureus* isolated from a patient	Right femurs	Bone_*S. aureus*	6	6	PRJNA701190	Lin, et al., 2021	(35)
*15*	*Citrobacter rodentium* DBS100	Liver	Liver_*C. rodentium*_DBS100	3	6	PRJNA435929	Sanchez, et al., 2018	(36)
*16*	*Escherichia coli* O55:B5, ATCC 12014	Liver	Liver_*E. coli*_ATCC120104	4	4	PRJNA506211	Li, et al., 2018	(37)
*17*	*Cryptococcus neoformans* var. grubii H99	Brain	Brain_*C. neoformans*_H99	3	3	PRJNA506308	Li, et al., 2019	(31)
*18*	*Mycobacterium tuberculosis* BJN	Raw264.7 macrophages	Mapha_ *M. tuberculosis* _BJN	4	4	PRJNA636677	Laopanupong, et al., 2021	(38)
*19*	A/California/04/09 *H1N1, -ssRNA*	Lung	*H1N1*	3	15	PRJNA385346	Forst, et al. 2022	(39)
*20*	A/Wyoming/03/03 *H3N2, -ssRNA*	Lung	*H3N2*	3	15	PRJNA385346	Forst, et al. 2022	(39)
*21*	A/Vietnam/1203/04 *H5N1, -ssRNA*	Lung	*H5N1*	3	15	PRJNA385346	Forst et al. 2022	(39)
*22*	*SARS-CoV-2, +ssRNA*	Lung	*SARS-CoV-2*	9	9	PRJNA805187	Tang, et al., 2022	(8)

-ssRNA, negative-stranded RNA; +ssRNA, positive-stranded RNA.

### Consensus signatures of A-to-I RNA editing in IBP infections

**Figure 2:**
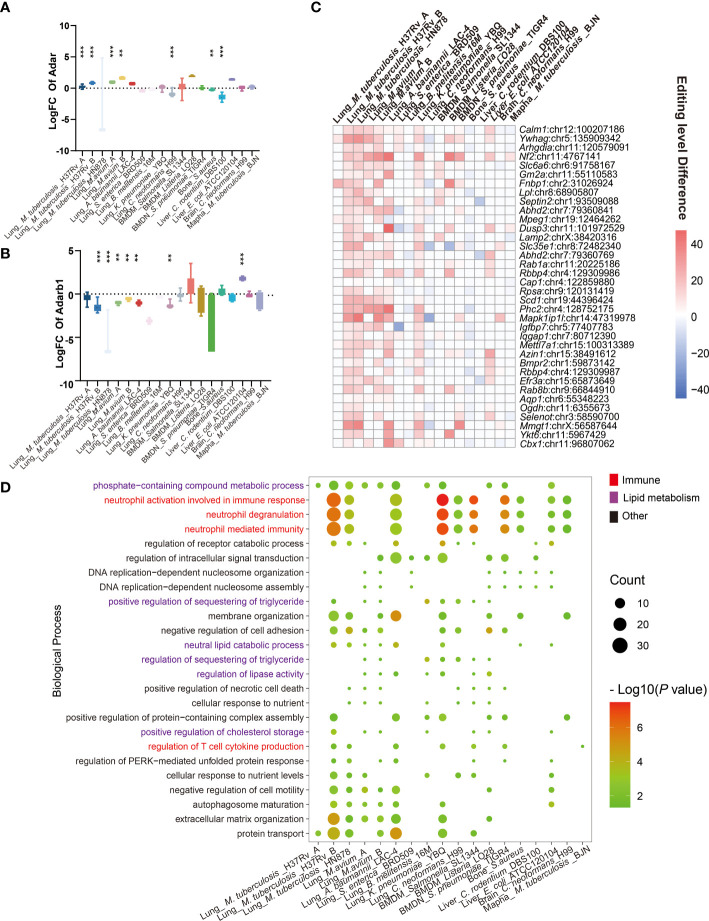
Consensus signature of A-to-I RNA editing in IBP infections. **(A)** The expression level differences of **(A)** *Adar* and **(B)** *Adarb1*, are shown as log2 (Fold changes) (the infected/uninfected). **(C)** Heatmap of DRE sites shared by at least seven IBP infection datasets. The color of the squares represents the editing level difference of edited sites between the infected and uninfected samples). **(D)** The significance of biological processes enriched by DRE genes for each infection dataset is represented by the point size. (log10 (*P* value)). Terms significantly enriched in at least seven IBP infection datasets are shown. The texts of immune-related items are colored in red. (The *Student*’s t-test is used for the inter-group comparisons of *Adar* and *Adarb1* expression; **P < 0.01; ***P < 0.001).

A-to-I RNA editing is mediated by RNA editing enzymes Adar and Adarb1 (13). Our results showed that *Adar* expression increased, while *Adarb1* decreased in most IBP infections (**Figures 2A, B**). DRE sites in all infections were further compared to identify shared DRE sites (**Supplementary Table S1** and **Figure 2C**). In particular, DRE sites in Calmodulin 1 (*Calm1*: chr12: 100207186) and Tyrosine 3-Monooxygenase/Tryptophan 5-Monooxygenase Activation Protein Gamma (*Ywhag*: chr5: 135909342) were shared by 12 infection datasets, which were also predicted to exert a cis-regulatory effect on the gene expression (**Supplementary Figures S2A, B**). Gene ontologies (GO) showed that the DRE genes were enriched in immune response pathways, such as neutrophil-mediated immunity and regulation of T cell cytokine production (**Supplementary Table S3**.), phosphate-containing metabolic compound process, lipid metabolism, and translational regulation (**Figure 2D**). The KEGG pathway analysis demonstrated enrichment of the DRE genes in lysosomes and autophagy pathways (**Supplementary Figure S2C**).

### RNA editing alteration in IBP pneumonia

**Figure 3:**
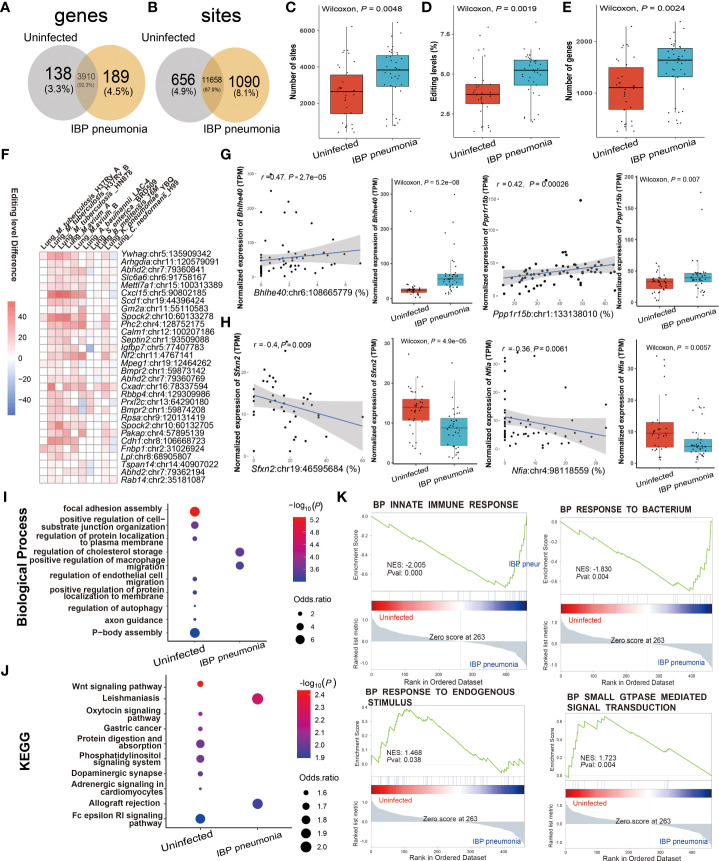
RNA editing alteration in IBP pneumonia. **(A)** Venn plot comparing the genes with A-to-I editing between the combined infected and uninfected groups. **(B)** Comparison of A-to-I RNA editing sites between the combined infected and uninfected groups. **(C)** The number and **(D)** editing level of A-to-I RNA editing sites, and **(E)** the number of A-to-I RNA editing genes. **(F)** Top 30 DRE sites shared by at least six pneumonia datasets. Each square represents the difference in the editing level of the edited site (the infected - the uninfected, all GLM test *P* < 0.05). **(G, H)** Spearman correlation between gene expression and editing level of *Bhlhe40, Ppp1r15b*, *Sfxn2*, and *Nfia*. Items with the most significant *P*-values are shown for **(I)** biological processes and **(J)** KEGG pathways. Selected GSEAs of DRE genes are listed in **(K)** GLM, general linear model.

Given the high incidence, infectivity, harm (40), and commonality of pneumonia, we further focused on its RNA editing. A-to-I RNA editing was the most frequent among all RNA editing types in terms of both editing sites and edited genes in IBP pneumonia (**Supplement Figure S1**). Thus we focused on A-to-I RNA editing in subsequent analysis. 656 editing sites in 138 edited genes and 1090 editing sites in 189 edited genes were exclusively detected in uninfected and infected lung tissues of the IBP pneumonia datasets, respectively (**Figures 3A, B**). Moreover, the number of RNA editing sites and edited genes as well as the editing level showed an up-regulated trend after bacterial infection (**Figures 3C–E**). More specifically, the top 30 sites that were the most differentially edited in IBP pneumonia datasets were shown in **Figure 3F**. As shown in **Figure 3G**, two significant cis-regulatory DRE sites with up-regulated RNA editing levels in infected lung tissues were found in Basic Helix-Loop-Helix Family Member E40 (*Bhhe40*) (*Bhlhe40*: chr6:108665779, Spearman *r* = 0.47, *P* = 2.7 × 10^−5^) and Protein Phosphatase 1 Regulatory Subunit 15B (*Ppp1r15b*) (*Ppp1r15b*: chr1:133138010, Spearman *r* = 0.42, *P* = 2.6 × 10^−4^). Two cis-regulatory DRE sites with down-regulated RNA editing levels were Sideroflexin 2 (*Sfxn2*) *(Sfxn2*: chr19:46595684, Spearman *r* = -0.4, *P* = 0.009) and Nuclear Factor I A (*Nfia*) *(Nfia*: chr4:98118559, with Spearman *r* = -0.36, *P* = 0.006) (**Figure 3H** and **Supplementary Table S4**). Enrichment analysis and Gene Set Enrichment Analysis (GSEA) revealed that these DRE genes were enriched in functions and pathways related to lipid metabolism, innate immunity, and GTPase-related regulation in IBP pneumonia (see **Figures 3I–K**).

### RNA editing profiles of ssRNA viral pneumonia

**Figure 4:**
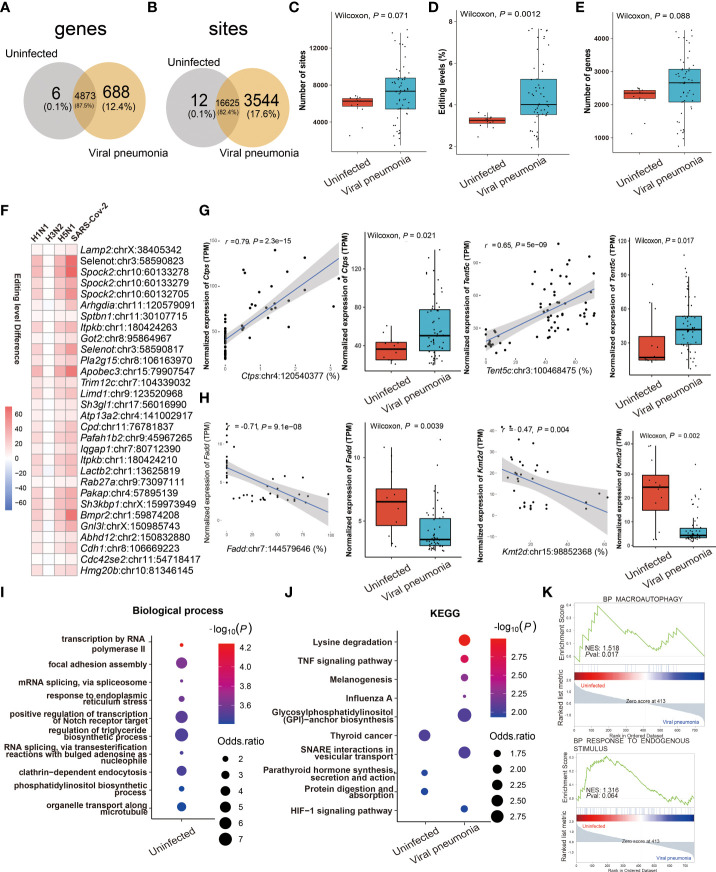
RNA editing profiles of ssRNA viral pneumonia. **(A)** Venn plot comparing the A-to-I editing sites detected in two or more samples among the groups. **(B)** Comparison of A-to-I RNA editing sites between the groups. **(C)** The number and **(D)** editing level of A-to-I RNA editing sites, and **(E)** the number of A-to-I RNA editing genes. **(F)** Top 30 DRE sites that shared by all ssRNA viral pneumonia. Each square represents the difference in the editing level of the edited site between uninfected and infected groups. **(G, H)** Spearman correlation between the gene expression and editing level of *Ctps, Tent5c*, *Fadd*, and *Kmt2d*. The most significantly enriched items of **(I)** biological processes and **(J)** KEGG pathway are shown. Selected GSEAs of DRE genes are listed in **(K)**.

Comparing the similarities and differences in RNA editing between viral and IBP pneumonia, A-to-I RNA editing was the most frequent among all RNA editing types (**Supplementary Figures S1C, D**), most of which were located in the 3'UTR (**Supplementary Figure S3D**). The RNA editing profiles of most viral pneumonia were similar to those in IBP pneumonia, with *Adar* up-regulated and *Adarb1* down-regulated (**Supplementary Figures S3A, B**). The Venn plots showed 12 editing sites in 6 edited genes and 3544 editing sites in 688 edited genes exclusively present in uninfected and infected lung tissues, respectively (**Figures 4A, B**). Likewise, the editing level, the number of editing sites, and genes were increased after viral infection (**Figures 4C–E**). However, *Adar* in *H3N2* infection was down-regulated (**Supplementary Figures S3E, F–H**), which was consistent with its overall changes in editing sites and levels (**Supplementary Figures S3F–H**). Notably, numerous shared sites were found among viral pneumonia datasets (**Figure 4F**). As shown in **Figure 4G**, two significantly cis-regulatory DRE sites with up-regulated RNA editing levels in the infected group were found in CTP synthase 1 (*Ctps*) (*Ctps*: chr4:120540377, Spearman *r* = 0.79, *P* = 2.3 × 10^−15^) and Terminal Nucleotidyltransferase 5C (*Tent5c*) (*Tent5c*: chr3:100468475, *Spearman r* = 0.65, *P* = 5.0 × 10^−9^). Two cis-regulatory DRE sites with down-regulated RNA editing levels were also observed in Fas Associated *Via* Death Domain (*Fadd*) *(Fadd*: chr7:144579646, Spearman *r* = - 0.71, *P* = 9.1× 10^−8^) and Lysine Methyltransferase 2D (*Kmt2d*: chr15:98852368, coefficient *r* = - 0.47, *P* = 4× 10^−3^) (**Figure 4H**). Enrichment analysis and GSEA showed that these DRE genes were mainly involved in the regulation of the triglyceride biosynthetic process, TNF signaling pathway, influenza A, and response to endogenous stimulus (**Figures 4I–K**).

### Comparison of RNA editing profiles between IBP and viral pneumonia

**Figure 5:**
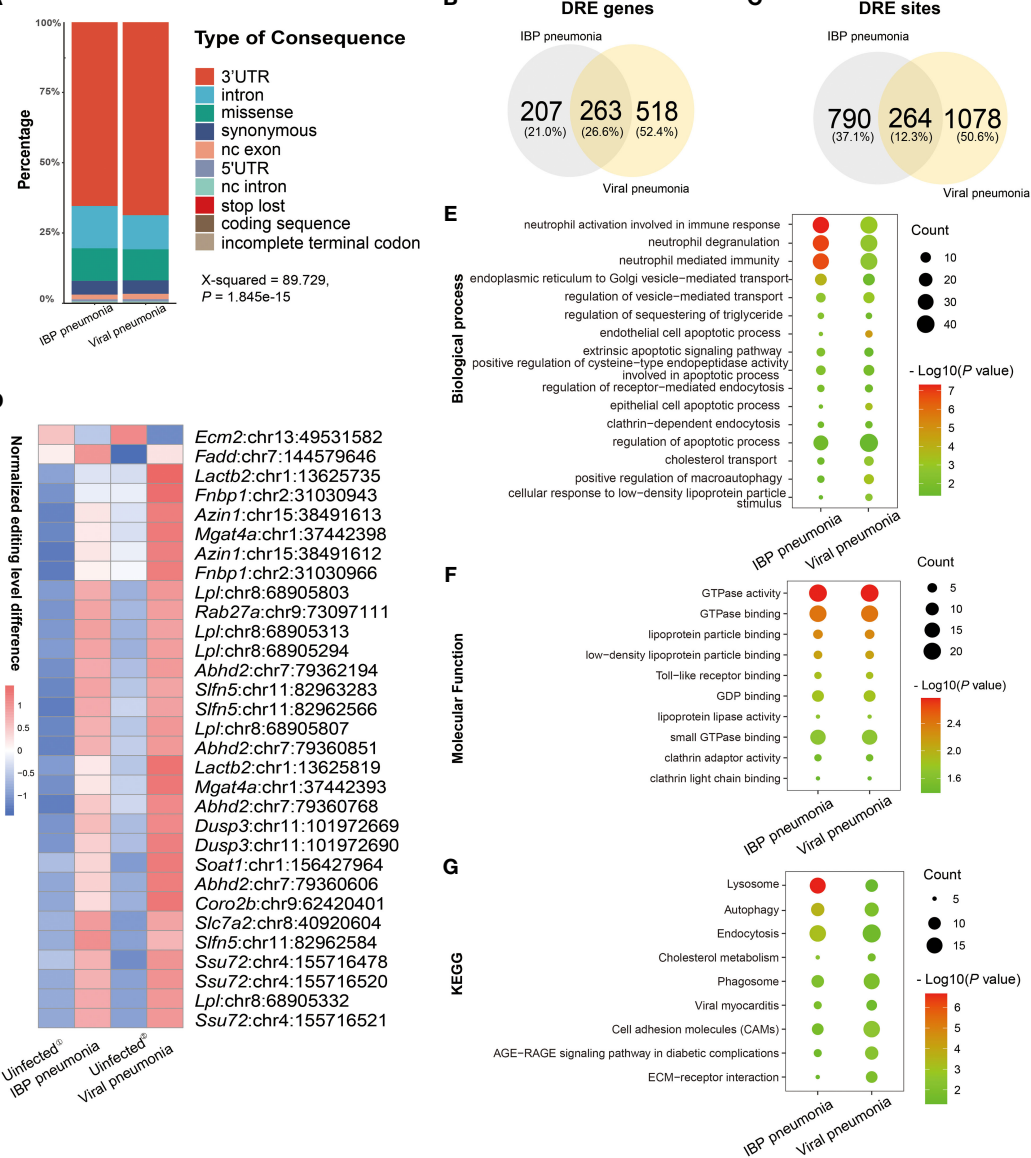
Comparison of RNA editing profiles between IBP and viral pneumonia. **(A)** The functional categories of A-to-I RNA editing sites in IBP and viral pneumonia. Venn plots comparing **(B)** differential RNA editing (DRE) genes and **(C)** sites between IBP and viral pneumonia. **(D)** Top 30 DRE sites shared by IBP and viral pneumonia. ①: controls for IBP; ②: controls for viruses. Shared **(E)** biological processes, **(F)** molecular functions, and **(G)** KKEGG pathways enriched by DRE genes between IBP and viral pneumonia.

By comparing IBP and viral pneumonia, we found that RNA editing in viral infections led to a higher proportion of 3′-UTR variants (**Figure 5A**). And most of the DRE sites were unique to IBP or viral pneumonia (**Figures 5B, C**). Interestingly, Spearman correlation analysis revealed more DRE sites were correlated with *Adar* compared to *Adarb1* in both IBP and viral infections (**Supplementary Figure S4**). Some DRE sites changed consistently in terms of editing level between the two types of infections (**Figure 5D**), which might further regulate gene expression. In line with this, similar up- or down-regulation in gene expression found in these DRE genes suggested mechanisms common to both types of infections (**Table 2**). Most of the shared DRE genes were hyper-edited. For example, Schlafen 5 (*Slfn5*) contained six A-to-I RNA editing sites (*Slfn5*: chr11:82962566, 82963283, 82963634, 82963686, 82962655 and 82962584). The shared GO and KEGG pathways enriched by DRE genes between IBP and viral infections were mainly related to myelocyte-mediated immunity, autophagy, apoptosis, lysosomes, and small GTPases. (**Figures 5E-G** and **Supplementary Tables S6**-**S9**). Therefore, such findings showed RNA editing changes shared by the two types of infections.

**Table 2:** Ten genes with the largest shared counts of DRE sites between IBP and viral infection models.

NO.	Genes	Number of shared DRE sites	With significantly differential gene expression between the uninfected and infected	
			IBP (LogFC)	Viral infection (LogFC)
1	*Slfn5*	6	0.384*	0.760**
2	*Ssu72*	4	0.571***	0.258**
3	*Soat1*	4	0.257*	0.490*
4	*Sppl2a*	2	0.950***	0.699**
5	*Rab27a*	1	0.485***	0.405***
6	*Sirpb1c*	1	1.860***	1.782***
7	*Coro2b*	1	-1.069***	-1.346***
8	*Dcp2*	1	0.464***	0.533***
9	*Ppp1r15b*	1	0.649***	0.306**
10	*Plekhd1*	1	-0.906***	-0.412*

GLM was used for the analysis of DRE genes; DRE: differential RNA editing; IBP: intracellular bacterial pathogens; *: P < 0.05; **: P < 0.01; ***: P < 0.001.

### DRE signatures distinguished IBP and viral pneumonia

**Figure 6:**
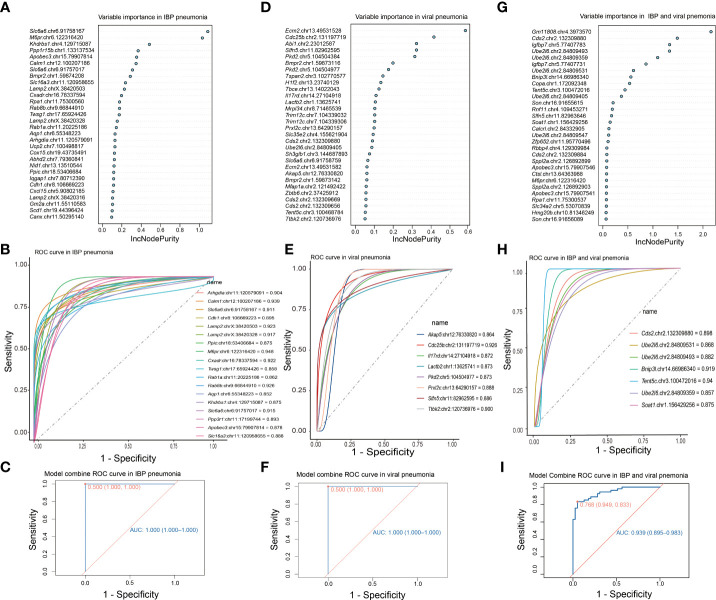
DRE signatures distinguish IBP and viral pneumonia. **(A)** The top 30 significant DRE sites identified by random forest in IBP pneumonia. **(B)** 19 DRE sites in IBP pneumonia with AUC greater than 0.85. **(C)** The ROC curve obtained from logistic regression of these 19 sites. **(D)** Random forest identifies the top 30 significant DRE sites in viral pneumonia **(E)** and 8 DRE sites in viral pneumonia with AUC greater than 0.85. **(F)** The ROC using the combination of the above 8 DRE sites. **(G)** Random forest identifies the top 30 significant DRE sites shared by IBP and viral pneumonia. **(H)** 7 DRE sites in IBP and viral pneumonia with AUC greater than 0.85. **(I)** The ROC curve using the combination of the above 7 DRE sites. The top 30 sites are ranked by their contribution to the Increase in Node Purity (IncNodePurity). ROC, receiver operating characteristic; AUC, the area under the ROC curve.

RNA editing could be used in the diagnosis of diseases (41, 42). Therefore, we focused on the change of RNA editing profiles that have certain consensus and specificity, which were used for the diagnosis of related infectious disease models. To determine DRE sites with diagnostic significance, we first performed random forest analysis of the identified DRE sites and obtained the top 30 significant DRE sites (**Figures 6A, D**). We selected those sites that were only present in either IBP or viral pneumonia for receiver operating characteristic curve (ROC) analysis, kept those with area under curve (AUC) > 0.85 for linear regression analysis (**Figures 6B, E**) and obtained two diagnostic curves for the two types of infections, respectively (**Figures 6C, F**). Further comparison of these DRE sites between IBP and viral infections obtained seven sites with AUC > 0.85, which were included in a diagnostic model to predict the type of infections (**Figures 6G**– **I**). And the results showed that combined analysis of these sites had high sensitivity and specificity in distinguishing IBP infections from viral infections.

## Discussion

Although IBP infections have been reported to lead to transcriptome remodeling of immune functions similar to viral infections (9, 10), the role of RNA editing in this process has not been investigated systematically. The current study presented the first systematic characterization of host RNA editing alterations in IBP infections.

It had been reported that the increased RNA editing events were accompanied by the up-regulation of the editing enzyme *Adar* expression in influenza and fungus-infected host cells (18, 19). Our results observed similar altered expression of *Adar* and *Adarb1* in most of the IBP infections. Recent studies proposed that innate immune responses to polyomavirus infection in mice were regulated by Adar, but not Adarb1 (43). Given the strong correlation between Adar expression and A-to-I RNA editing in our results, we thus speculated that Adar could be the main RNA editing enzyme involved in the process of IBP or viral infections, which was consistent with Ward’s report that Adar P150 was a limiting factor for influenza A virus replication (44).

Furthermore, a large number of potential cis-regulatory RNA editing sites were found in the 3’-UTR. Among them, *Bhlhe40* was reported to participate in macrophage-mediated immunity (45), *Sfxn2* acted as a key gene regulating mitochondrial iron homeostasis in cells (46), and knockdown of *Nfia* was reported to promote cell adhesion of *Klebsiella pneumoniae* (47). For the genes with cis-regulatory DRE in viral infections, *Ctps* and *Tent5c* served as the critical signal factors in lymphocyte proliferation (48, 49) and Kmt2d regulates CD8 T cell development and differentiation (50). Such findings could thus underscore the role of cis-regulatory A-to-I RNA editing in the immune response to intracellular infections.

In addition, the functional analysis revealed that apoptosis-related pathways such as endothelial cell apoptosis and regulation of the apoptotic process were involved in RNA editing changes common to both types of infections (**Figure 5E**). Apoptosis genes *Calm1*, *Ywhag*, and *Ppp1r15b* showed up-regulated RNA editing in IBP infections (**Figures S2A, B**) (51, 52). *Fadd* -mediated apoptosis was a pivotal pathway against viral infections (53). Additionally, *Slfn5*, with shared DRE sites between IBP and viral infections (**Table 2**), inhibited apoptosis by regulating the mTOR pathway (54). These results suggested that RNA editing may affect host cell apoptosis after infection.

The autophagolysosomal pathway was altered in both IBP and viral infections, which acts as a highly conserved intracellular degradation pathway in eukaryotes (55). It is involved in pathogen removal (56). Consistently, up-regulation of DRE genes associated with the autophagolysosomal pathway such as *Rab27a (Rab27a:chr9:73097111*) (**Figure 5D** and **Table 2**) was found in both IBP and viral infections in the current study. Moreover, the Rab GTPase related functions in both IBPs and ssRNA viral infections were also altered. More specifically, the Rab GTPase family has also been demonstrated to be involved in the formation of autophagosomes and trafficking to lysosomes in bacterial and viral entry into host cells (57, 58). Recent studies also confirmed that the GTPase activity could promote antimicrobial immunity, targeting intracellular pathogens through inflammasomes and autophagy to mediate host defense responses (59–62).

Furthermore, the pathways of DRE genes identified in IBP and viral infections were mostly related to neutrophils (**Figures 2D**, **5E**), which was in line with the reported high influx of neutrophils infiltrating into infected sites to remove pathogens (63, 64). Recent studies have also confirmed that Adar deficiency leads to impaired development of neutrophils (65). Therefore, A-to-I RNA editing mediated by Adar might be associated with neutrophil-mediated immunity during IBP and viral infections.

In addition, it was noted IBPs and viruses may influence host regulation of lipid metabolism *via* RNA editing (**Figures 2D**, **3J**, **5G**), which may be involved with pathogen-host interactions (66). For instance, the main nutrient source of some IBPs such as Mycobacterium tuberculosis in the host was lipids from the cytosol (67). Lipids can also promote the replication of SARS-CoV-2 and the production of inflammatory mediators (68), which suggests that changes in host lipid-related functions may affect the viability of IBPs and viruses. Accordingly, DRE sites in lipid-related genes such as Lipoprotein lipase (Lpl) and CDP Diacylglycerol Synthase 2 (Cds2) (**Figure 2D**) were upregulated after infection (69–71).

Meanwhile, recent studies have indicated the application of RNA editing in the diagnosis of diseases such as cancers (41, 42). Our work highlights the RNA editing difference between IBP and viral infections, particularly for genes involved in immune responses. For example, Cell Division Cycle 25B (*Cdc25b*), a key factor for virus replication (72), showed DRE in viral infections only but not in bacterial infections. The developed diagnostic method based on these type-specific sites (**Supplementary Tables S4**, **5**) could be used to distinguish IBP and viral infections (**Figure 6I**).

In general, our study showed similarity in host A-to-I RNA editing signatures in IBPs and ssRNA viral infections, suggesting underlying common potential pathogen-host interaction. The A-to-I RNA editing associated with IBP and viral infections could provide new insight into the identification of novel diagnostic and therapeutic targets. Further study will be needed to investigate the biological effects of RNA editing on the edited genes and downstream pathways at the RNA and protein levels, especially those with hyper-editing in the interaction between the pathogens and the host.

## Materials and methods

### RNA-seq datasets

Raw data of RNA-Seq were downloaded from the European Nucleotide Archive (ENA) of the European Molecular Biology Laboratory (https://www.ebi.ac.uk/ena). The details of all bacterial and viral infection datasets can be accessed in **Table 1**.

### Alignment of RNA sequencing data

The process of RNA sequencing reads was conducted as previously described (73). In brief, the raw sequencing data analyzed by FastQC for quality control were aligned and mapped to the mouse genome (UCSC mm10) using RNA STAR (version 2.7.0e) (74). SamTools (version 1.16) was used to filter the reads by removing optic duplications (75), and only reads uniquely mapped were kept. Base quality score recalibration was then performed with the resulting BAM files by using GATK (version 4.1.3) and following the best practice workflows recommended by the documentation (76).

### Identification of high-confidence A-to-I RNA editing

Single nucleotide variants (SNV) were called by using VarScan (version 2.4.4) (77). The variant calling criteria were set as follows: base quality ≥25, total sequencing depth ≥10, alternative allele depth ≥2, and alternative allele frequency (AAF) ≥1%, and possible false positive SNVs were filtered and removed using VarScan with default parameters. SNVs were annotated using the Ensembl Variant Effect Predictor (VEP) (78). SNVs were further filtered and removed according to criteria described previously (73): (1) located in homopolymer runs ≥ 5 nucleotides (nt), simple repeats, in the mitochondria, within 6 nt from splice junctions, within 1 nt from insertions or deletions, or within 4% to the ends of reads; (2) annotated in the dbSNP database Build 142 unless annotated as RNA editing sites in the REDIportal V2.0 database (79) (3); more than 90% of all samples had an AAF equal to 100% or between 40% and 60% (80). High-confidence A-to-I (G) RNA editing SNVs (including A-to-G genomic SNVs on the coding strand and T-to-C genomic SNVs on the opposite strand) were defined either as known RNA editing sites in the REDIportal V2.0 database, or located in genic regions and detected in at least 2 samples with editing levels ≥1%.

### Quantification of gene expression in RNA-seq

Pseudo-counts of gene expression were calculated from the RNA-Seq alignment files using FeatureCounts (81), and transcripts read per thousand bases per million mappings (TPM) were then obtained for each gene using edgeR (version 3.7) (82).

### Principal component analysis

Principal component analysis (PCA) was performed using the function prcomp in R (version 4.2.1) and visualized using the ggplot2 package (version 2.2.1). Heatmaps were plotted using the Pheatmap package in R (version 4.2.1).

### Random forest and ROC analysis

Random Forest (83) was used to identify RNA editing sites as biomarkers with high sensitivity and specificity for the diagnosis of infection types. The receiver operator characteristic curve analysis was performed and the area under the curve (AUC) was calculated using the pROC package of R (84).

### Enrichment analysis of gene function and pathways

The enrichment analysis of genes with RNA editing including gene ontology (GO) and Kyoto encyclopedia of genes and genomes (KEGG) pathways were conducted using Enrichr (85).

### Enrichment analysis by gene set enrichment analysis

The GSEA version 4.2.3 software and dataset were used to function of genes based on the GSEA website MSIGDB database (https://www.gsea-msigdb.org/gsea/msigdb/mouse_geneset_resources.jsp) (86), using a default weighted enrichment method with 1000 permutations. Enrichment with false discovery rate (FDR) < 0.25, nominal *P*-value < 0.05, and |normalized enrichment score (NES)|> 1 were considered significant. NES indicated the analysis results across gene sets. Pairwise *P*-values were calculated using the non-parametric Kruskal-Wilcoxon test followed by the Tukey *post-hoc* test.

### Statistical analysis

The generalized linear model (GLM) method and likelihood ratio test were used to compare the intergroup RNA editing levels. The *Student*’s t-test was used to compare gene expression levels. The *Spearman* correlation was used to analyze the correlation between RNA editing and gene expression, and correlation coefficients (*r*) and *P*-values were calculated. The statistical significance level was set at *P* < 0.05.

## Data availability statement

The original contributions presented in the study are included in the article/**Supplementary Material**. Further inquiries can be directed to the corresponding authors.

## Author contributions

Conceptualization: Z-YW, Z-XW, J-HC, Y-YJ, Y-SL, Data curation: Z-YW, Z-XW. Funding acquisition: J-HC, Y-YJ. Investigation: Z-YW, Z-XW, Y-SW, DG, S-YX, J-HL. Methodology: Z-YW, Z-XW, Y-SW, DG, Y-NL, J-HL. Supervision: J-HC, Y-YJ, Y-SL, X-BP. Writing – original draft: Z-YW, Z-XW. Writing – review & editing: Z-YW, Z-XW, J-HC, Y-YJ. All authors contributed to the article and approved the submitted version.
