# Supplementary material for: Host A-to-I RNA editing signatures in intracellular bacterial and single-strand RNA viral infections
Source: Front Immunol. 2023 Apr 4;14:1121096. doi: 10.3389/fimmu.2023.1121096 (PMC10112020; doi:10.3389/fimmu.2023.1121096)
Supplement: Supplementary file 1 [file Image_1.pdf]

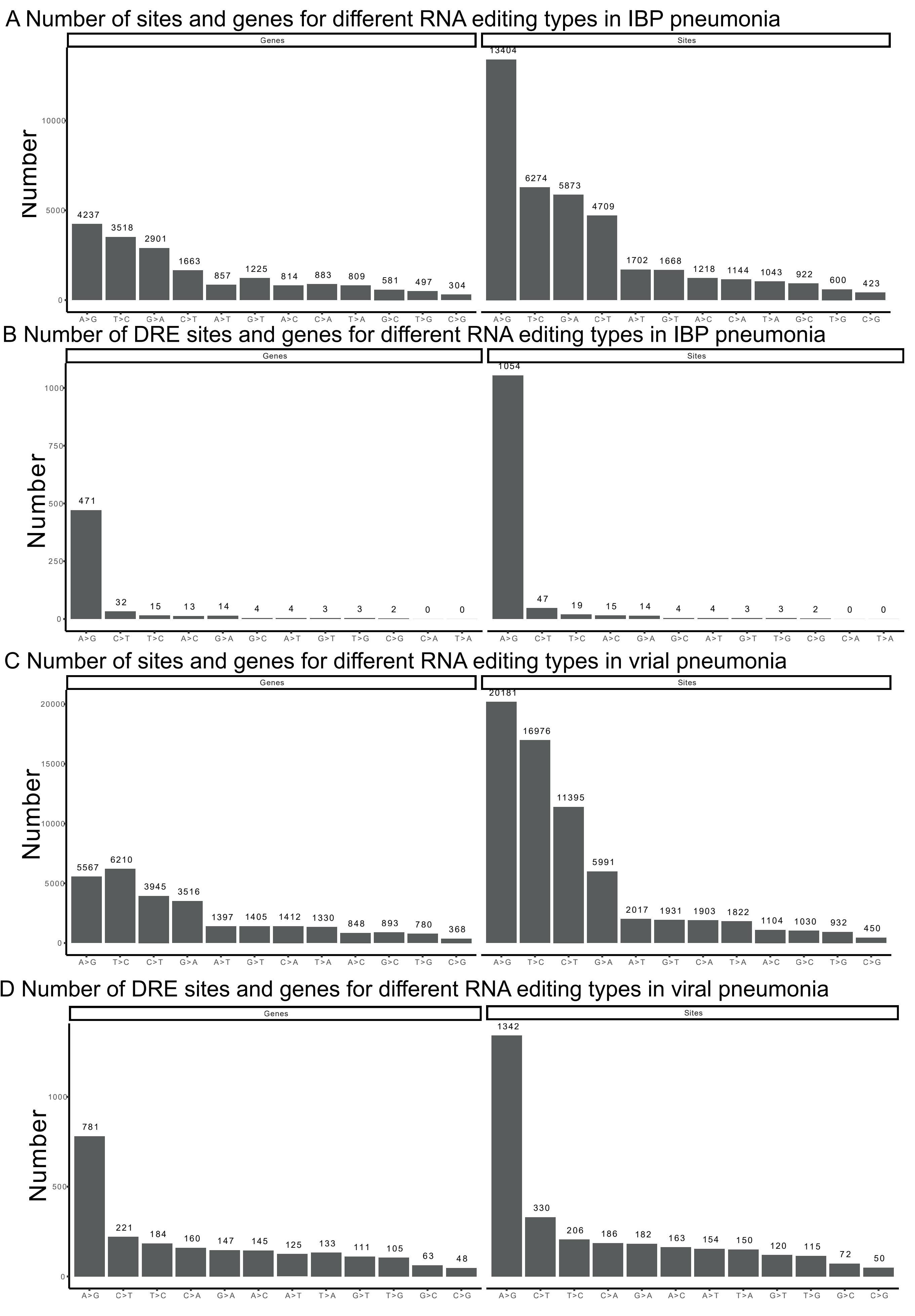
A Number of sites and genes for different RNA editing types in IBP pneumonia
Genes
Sites
Number
0
5000
10000
4237 3518 2901 1663 857 1225 814 883 809 581 497 304
A>G T>C G>A C>T A>T G>T A>C C>A T>A G>C T>G C>G
13404 6274 5873 4709 1702 1668 1218 1144 1043 922 600 423
A>G T>C G>A C>T A>T G>T A>C C>A T>A G>C T>G C>G
B Number of DRE sites and genes for different RNA editing types in IBP pneumonia
Genes
Sites
Number
0
250
500
750
1000
471 32 15 13 14 4 4 3 3 2 0 0
A>G C>T T>C A>C G>A G>C A>T G>T T>G C>G C>A T>A
1054 47 19 15 14 4 4 3 3 2 0 0
A>G C>T T>C A>C G>A G>C A>T G>T T>G C>G C>A T>A
C Number of sites and genes for different RNA editing types in vrial pneumonia
Genes
Sites
Number
0
5000
10000
15000
20000
5567 6210 3945 3516 1397 1405 1412 1330 848 893 780 368
A>G T>C C>T G>A A>T G>T C>A T>A A>C G>C T>G C>G
20181 16976 11395 5991 2017 1931 1903 1822 1104 1030 932 450
A>G T>C C>T G>A A>T G>T C>A T>A A>C G>C T>G C>G
D Number of DRE sites and genes for different RNA editing types in viral pneumonia
Genes
Sites
Number
0
500
1000
781 221 184 160 147 145 125 133 111 105 63 48
A>G C>T T>C C>A G>A A>C A>T T>A G>T T>G G>C C>G
1342 330 206 186 182 163 154 150 120 115 72 50
A>G C>T T>C C>A G>A A>C A>T T>A G>T T>G G>C C>G
