# Supplementary figures and images for: Host A-to-I RNA editing signatures in intracellular bacterial and single-strand RNA viral infections
Source: Front Immunol. 2023 Apr 4;14:1121096. doi: 10.3389/fimmu.2023.1121096 (PMC10112020; doi:10.3389/fimmu.2023.1121096)

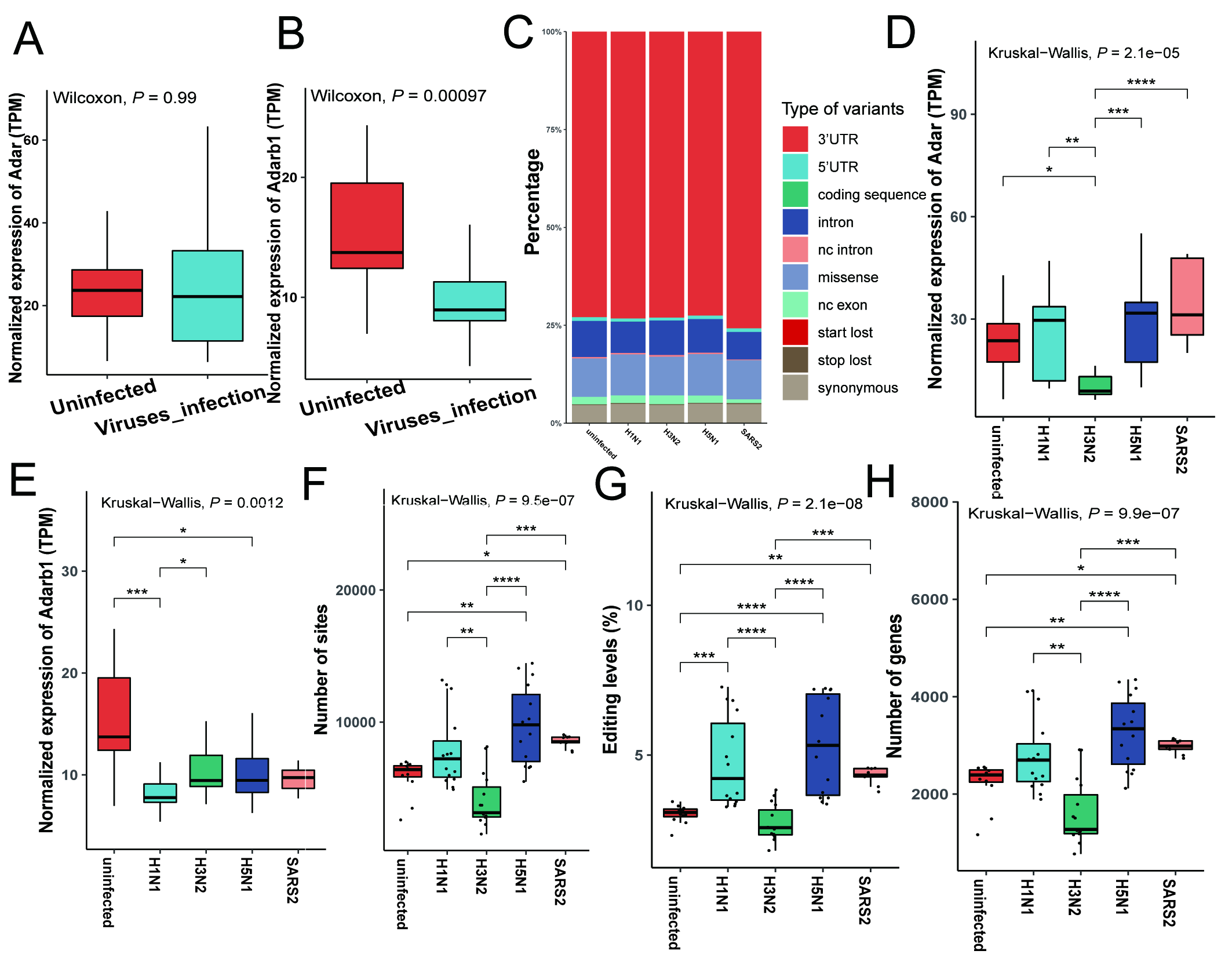

Supplement: Supplementary file 3 [file Image_3.tif]
