# Supplementary material for: Host A-to-I RNA editing signatures in intracellular bacterial and single-strand RNA viral infections
Source: Front Immunol. 2023 Apr 4;14:1121096. doi: 10.3389/fimmu.2023.1121096 (PMC10112020; doi:10.3389/fimmu.2023.1121096)

Correlation of DRE sites detected in **bacterial** infection models with gene (A) *Adar*, (B) *Adarb1*

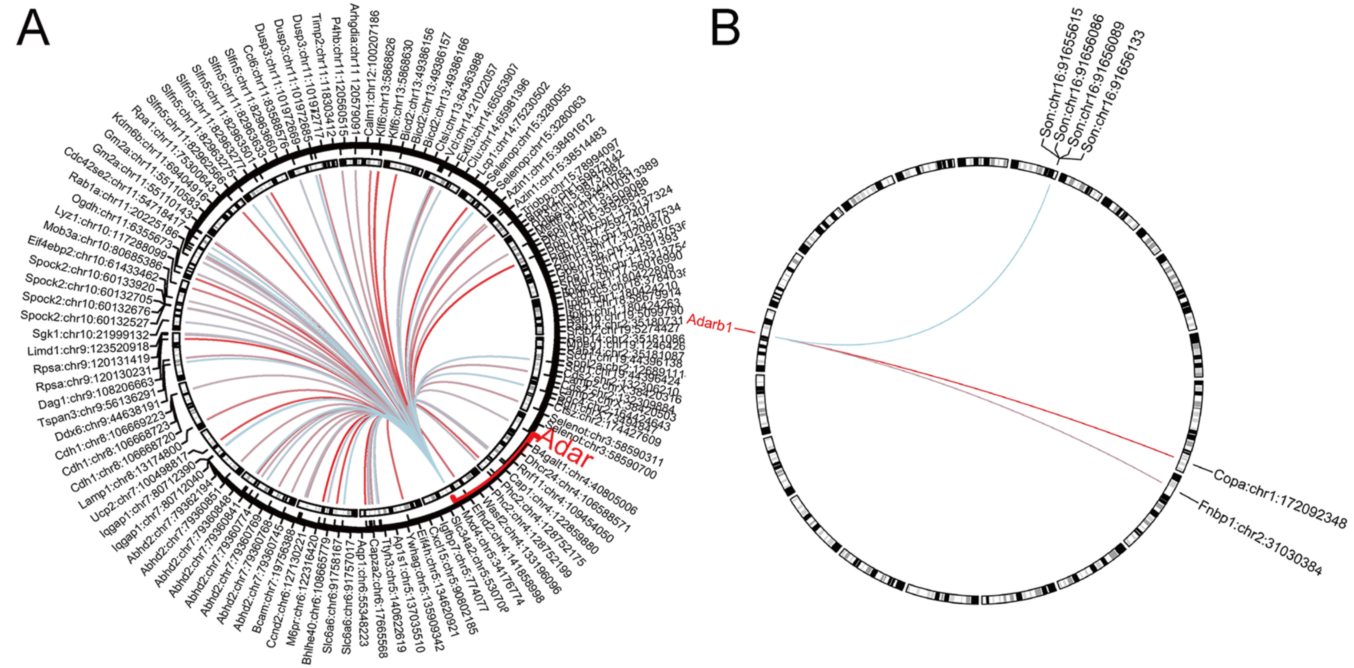

Correlation of DRE sites detected in **viral** infection models with gene (C) *Adar*, (D) *Adarb1*

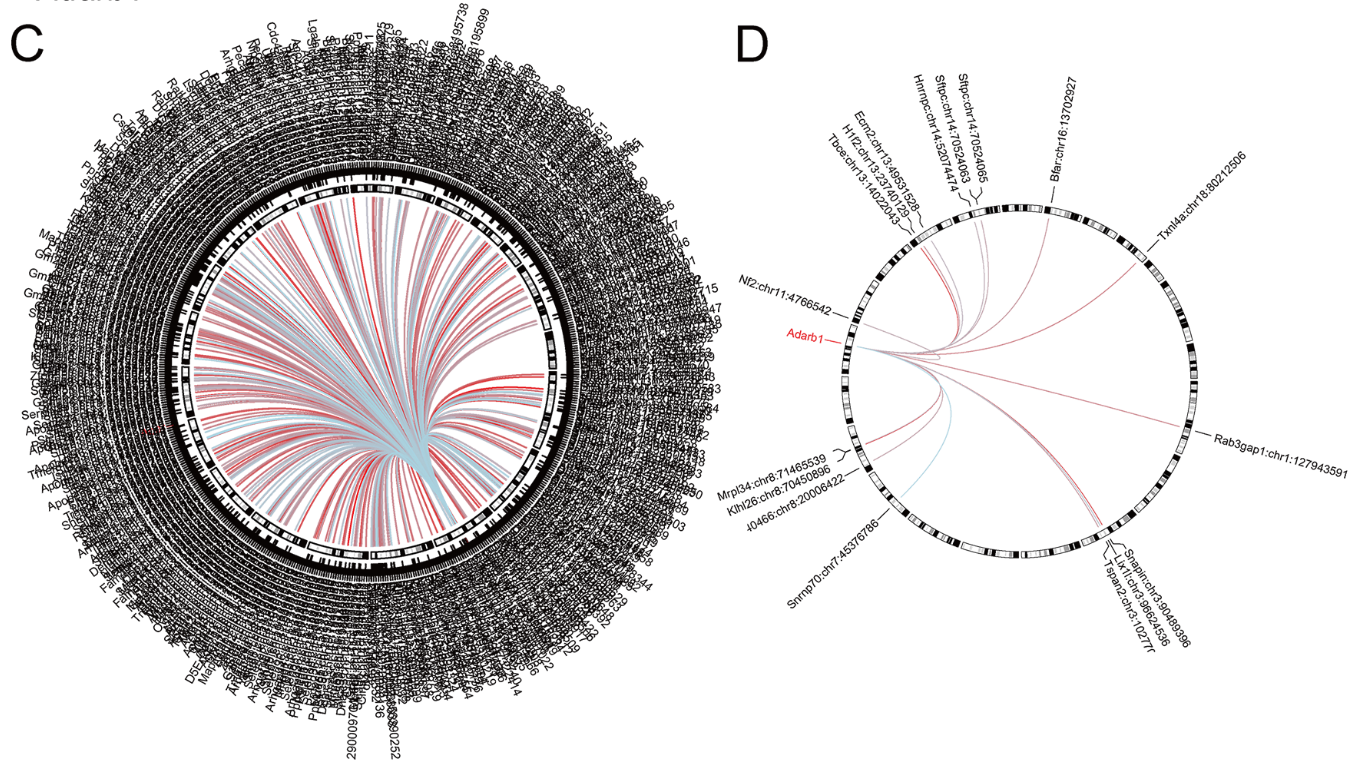

Supplement: Supplementary file 4 [file Image_4.pdf]
