# Supplementary material for: Host A-to-I RNA editing signatures in intracellular bacterial and single-strand RNA viral infections
Source: Front Immunol. 2023 Apr 4;14:1121096. doi: 10.3389/fimmu.2023.1121096 (PMC10112020; doi:10.3389/fimmu.2023.1121096)
Supplement: Supplementary file 2 [file Image_2.pdf]

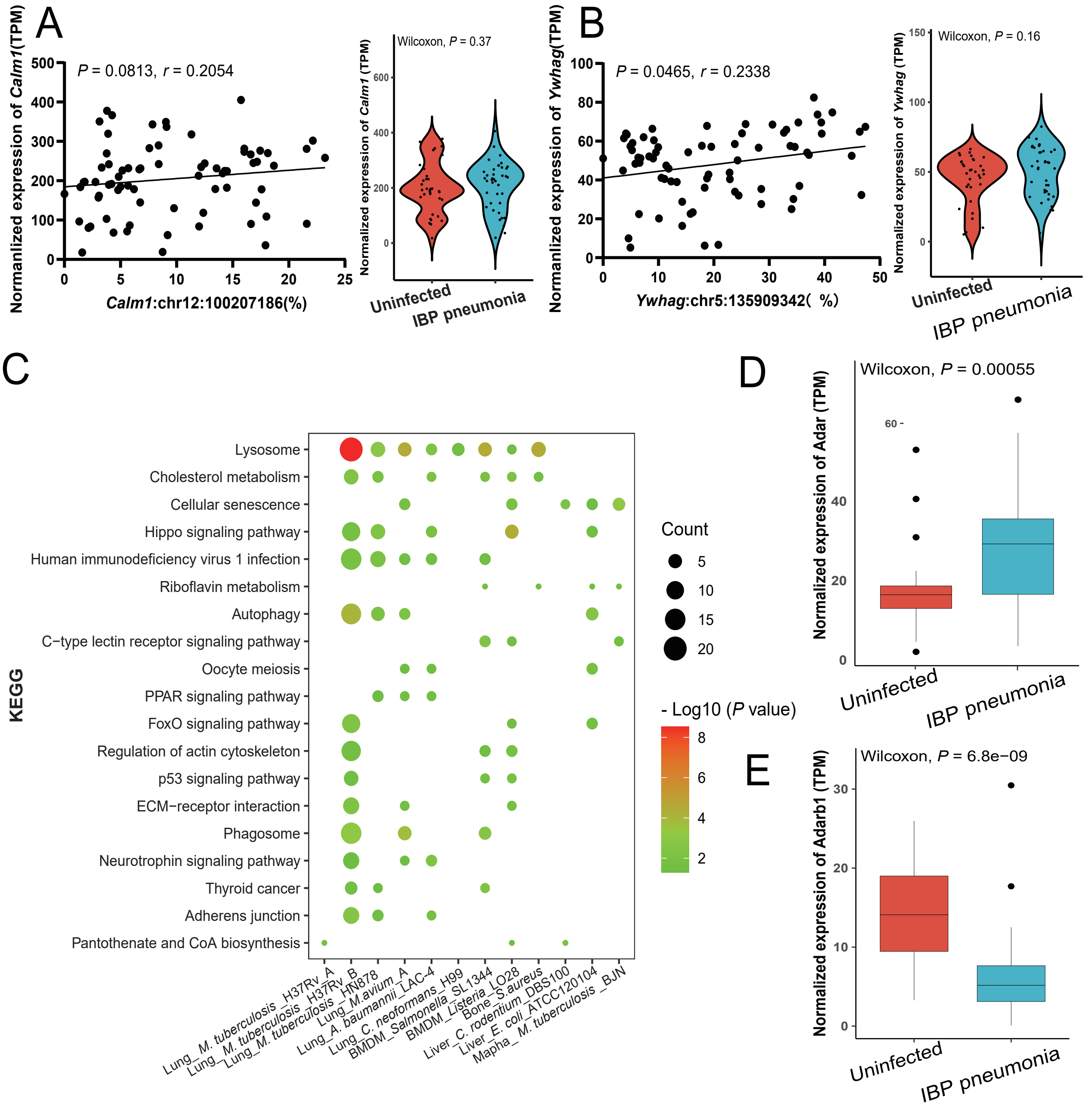

A
Normanlized expression of Calm1(TPM)
P = 0.0813, r = 0.2054
Calm1:chr12:100207186(%)
Wilcoxon, P = 0.37
Normalized expression of Calm1 (TPM)
Uninfected
IBP pneumonia
B
Normanlized expression of Ywhag(TPM)
P = 0.0465, r = 0.2338
Ywhag:chr5:135909342(%)
Wilcoxon, P = 0.16
Normalized expression of Ywhag (TPM)
Uninfected
IBP pneumonia
C
KEGG
Lysosome
Cholesterol metabolism
Cellular senescence
Hippo signaling pathway
Human immunodeficiency virus 1 infection
Riboflavin metabolism
Autophagy
C−type lectin receptor signaling pathway
Oocyte meiosis
PPAR signaling pathway
FoxO signaling pathway
Regulation of actin cytoskeleton
p53 signaling pathway
ECM−receptor interaction
Phagosome
Neurotrophin signaling pathway
Thyroid cancer
Adherens junction
Pantothenate and CoA biosynthesis
Lung_M. tuberculosis _H37Rv_A
Lung_M. tuberculosis _H37Rv_B
Lung_M. tuberculosis _HN878
Lung_M.avium_A
Lung_A. baumannii_LAC-4
Lung_C. neoformans_H99
BMDM_Salmonella_SL1344
BMDM_Listeria_LO28
Bone_S.aureus
Liver_C. rodentium_DBS100
Liver_E. coli_ATCC120104
Mapha_ M. tuberculosis _BJN
Count
5
10
15
20
- Log10 (P value)
8
6
4
2
D
Wilcoxon, P = 0.00055
Normalized expression of Adar (TPM)
Uninfected
IBP pneumonia
E
Wilcoxon, P = 6.8e−09
Normalized expression of Adarb1 (TPM)
Uninfected
IBP pneumonia
